# Regorafenib (Stivarga) pharmacologically targets epithelial-mesenchymal transition in colorectal cancer

**DOI:** 10.18632/oncotarget.11636

**Published:** 2016-08-26

**Authors:** Li-Ching Fan, Hao-Wei Teng, Chung-Wai Shiau, Wei-Tien Tai, Man-Hsin Hung, Shung-Haur Yang, Jeng-Kai Jiang, Kuen-Feng Chen

**Affiliations:** ^1^ Department of Medical Research, National Taiwan University Hospital, Taipei, Taiwan; ^2^ National Center of Excellence for Clinical Trial and Research, National Taiwan University Hospital, Taipei, Taiwan; ^3^ Division of Hematology and Oncology, Department of Medicine, Taipei Veterans General Hospital, Taipei, Taiwan; ^4^ Institute of Biopharmaceutical Sciences, National Yang-Ming University, Taipei, Taiwan; ^5^ Division of Medical Oncology, Department of Oncology, Taipei Veterans General Hospital, Taipei, Taiwan; ^6^ Division of Colon and Rectal Surgery, Department of Surgery, Taipei Veterans General Hospital, Taipei, Taiwan; ^7^ School of Medicine, National Yang-Ming University, Taipei, Taiwan

**Keywords:** regorafenib, EMT, SHP-1, STAT3, CRC

## Abstract

Epithelial-to-mesenchymal transition (EMT) is well-known to evoke cancer invasion/metastasis, leading to a high frequency of mortality in patients with metastatic colorectal cancer (mCRC). Protein tyrosine phosphatase (PTPase)-targeted therapy has been identified as a novel cancer therapeutic. Previously, we proved that sorafenib with anti-EMT potency prevents TGF-β1-induced EMT/invasion by directly activating SH2-domain-containing phosphatase 1 (SHP-1)-dependent p-STAT3^Tyr705^ suppression in hepatocellular carcinoma. Regorafenib has a closely related chemical structure as sorafenib and is approved for the pharmacotherapy of mCRC. Herein, we evaluate whether regorafenib activates PTPase SHP-1 in the same way as sorafenib to abolish EMT-related invasion/metastasis in CRC. Notably, regorafenib exerted potent anti-EMT activity to curb TGF-β1-induced EMT/invasion *in vitro* as well inhibited lung metastatic outgrowth of SW480 mesenchymal cells *in vivo*. Mechanistically, regorafenib-enhanced SHP-1 activity significantly impeded TGF-β1-induced EMT/invasion *via* low p-STAT3^Tyr705^ level as proved by a SHP-1 inhibitor or siRNA-mediated SHP-1 depletion. Conversely, overexpression of SHP-1 further enhanced the inhibitory effects of regorafenib on TGF-β1-induced p-STAT3^Tyr705^ and EMT/invasion. Regorafenib directly activates SHP-1 by potently relieving the autoinhibited N-SH2 domain of SHP-1 to inhibit TGF-β1-induced p-STAT3^Tyr705^ and EMT/invasion. Importantly, the clinical evidence indicated that SHP-1 was positively correlated with E-cadherin and that significantly determined the overall survival of CRC patients. This result further confirms our in *vitro* data that SHP-1 is a negative regulatory PTPase in EMT regulation and serves as a pharmacological target for mCRC therapy. Collectively, activating PTPase SHP-1 by regorafenib focusing on its anti-EMT activity might be a useful pharmacotherapy for mCRC.

## INTRODUCTION

Metastatic colorectal cancer (mCRC) is a significant cause of mortality and morbidity worldwide because of its high biological heterogeneity. Despite the promising outcome of targeted therapies for mCRC, including those using anti-EGFR antibodies, and VEGF inhibitors, the drug resistance to the primary and secondary therapy remains a clinical challenge in mCRC. Most notably, regorafenib (BAY 73-4506, commercial name Stivarga) was approved by the FDA in September 2012 and is used for mCRC patients as a last-line therapy. It is an oral multitargeted tyrosine kinase inhibitor (TKI) of BRAF, VEGFR-1, -2, -3, KIT, TIE-2, PDGFR-β, FGFR-1, RET and RAF-1 that is particularly involved in oncogenesis and angiogenesis [1, 2]. Evidence from the murine metastatic CRC model also indicates that regorafenib potently abolishes angiogenesis and metastasis in advanced CRCs [3]. Importantly, epithelial-to-mesenchymal transition (EMT) is well known to be associated with tumor invasion and metastasis, including mCRC [4–7]. Whether regorafenib exerts anti-metastatic activity is because of its capability to inhibit EMT in CRC is not fully understood and yet to be characterized.

Src homology region 2 (SH2) domain-containing phosphatase 1 (SHP-1), a non-receptor protein tyrosine phosphatase (PTP), has been identified as a key negative regulator of cytokine signaling and immune cell activation [8, 9]. Interestingly, it also shows significant preclinical antitumor activity by triggering apoptosis *in vitro* and suppressing tumor formation *in vivo via* the PTP activity of SHP-1 that negatively targets p-STAT3^Tyr705^ signals in various cancer types [10–13]. Of note, STAT3 signaling is not only important for cancer cells in antagonizing apoptosis but has also been reported to be associated with cancer EMT by STAT3 transcriptional activation of EMT inducers TWIST1 and ZEB1 involved in E-cadherin repression [14–17]. The available clinical evidence of p-STAT3^Tyr705^ in EMT progression also reveals a significant positive correlation with tumor, lymph node and metastasis stages (TNM) [17, 18]. Most notably, our team previously discovered that SHP-1 acts as a strong suppressor, preventing TGF-β1-induced EMT characteristics *in vitro* and inhibiting metastatic growth *in vivo* in hepatocellular carcinoma (HCC) by exerting tyrosine phosphatase activity that downregulates p-STAT3^Tyr705^ directly [14]. Moreover, sorafenib, a targeted therapeutic drug for HCC, has been identified as a SHP-1 agonist that increases SHP-1 activity directly through its docking potential to N-SH2 and the catalytic PTP domain of SHP-1, leading to the relief of autoinhibition of SHP-1 [14, 19]. This contributes to the depletion of p-STAT3^Tyr705^-mediated EMT characteristics induced by TGF-β1 [14]. Our findings therefore present a novel relationship between SHP-1 and HCC EMT and reveal that targeting of the SHP-1-STAT3 axis by sorafenib may be an attractive strategy for HCC patients with metastasis.

Regorafenib (Fluror-sorafenib) has a chemical structure that is closely related to sorafenib and is the first approved pharmacotherapy for mCRC patients who have exhausted current standard therapies. In this study, we clarify whether regorafenib potently inhibits CRC EMT and metastasis in the same way as sorafenib by directly targeting the SHP-1-STAT3 axis. The identification of SHP-1 as a suppressor of CRC EMT and a druggable target of regorafenib focusing on its anti-EMT activity will advance our knowledge regarding the novel relationship between regorafenib and anti-EMT potency and highlights a therapeutic strategy for mCRC centering on the negative regulatory PTPase SHP-1.

## RESULTS

### A novel link between the SHP-1-E-cadherin axis and overall survival of CRC patients

**Figure 1:**
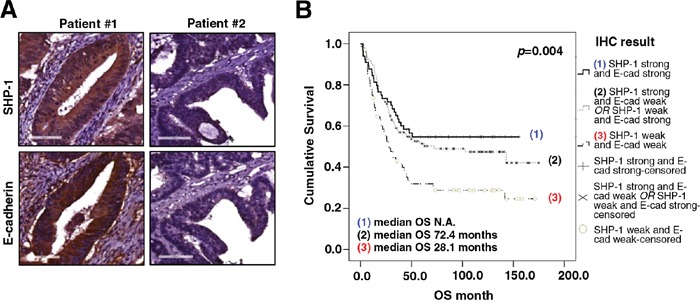
The expression of SHP-1 and E-cadherin correlates with overall survival of CRC patients **A.** Representative immunohistochemical patterns revealed that clinical CRC patient#1 with strong positive expression of SHP-1 had strong positive expression of E-cadherin. Patient#2 with weak positive expression of SHP-1 had weak positive expression of E-cadherin. **B.** Patients with weak expression of both SHP-1 and E-cadherin had a more significant reduction in median OS than others. (N.A., not available)

First, we used immunohistochemistry (IHC) to examine the expression level of SHP-1 and E-cadherin in CRC tissues and then investigated the clinical associations between SHP-1, E-cadherin and the clinicopathological parameters in CRC patients. 243 CRC patients were enrolled for this clinical analysis. As shown in Table 1, neither SHP-1 nor E-cadherin expression had a significant correlation with gender, AJCC stage, pathology or lymphovascular emboli. Moreover, there was no correlation between SHP-1 expression and histological grading. However, a significant inverse correlation was observed between E-cadherin and histological grading (*P* = 0.034), revealing that poorly differentiated human CRC with high histological grading showed loss of E-cadherin at cell boundaries, further suggesting the occurrence of EMT/metastasis. Most notably, we observed that a significant positive correlation existed between SHP-1 and E-cadherin expression (*P* = 0.038) (Table 1). In line with the results of statistical analysis, IHC patterns shown in CRC patient#1 with strong SHP-1 expression had strong E-cadherin expression. In contrast, patient#2 with weak SHP-1 expression had weak E-cadherin expression (Figure 1A). The overall survival (OS) curve performed by Kaplan-Meier analysis showed that no significant correlation between the median OS and SHP-1 expression (*P*=0.187) (Supplementary Figure 1). Notably, the median OS was not available in patients with strong expression both in SHP-1 and E-cadherin (Figure 1B). However, patients with weak expression of both SHP-1 and E-cadherin had a more significant reduction in median OS than others (*P*=0.004). These results suggest that SHP-1 is not an independent prognostic marker of CRC patients in our current study. Of note, patients with an elevated SHP-1-E-cadherin axis display longer survival. Taken together, targeting SHP-1-E-cadherin axis may be an effective treatment for suppressing EMT-related metastasis in CRC.

**Table 1:** The associations between SHP-1, E-cadherin and clinicopathological parameters were analyzed in 243 CRC patients

Clinicopathological parameters		SHP-1					E-cadherin				
		Weak		Strong			Weak		Strong		
		n	%	n	%	*P*	n	%	n	%	*P*
Gender	Female	40	36.0	44	33.6	*0.690*	45	33.8	39	35.8	*0.752*
	Male	71	64.0	87	66.4		88	66.2	70	64.2	
Location	Left	57	51.4	85	64.9	*0.033*	77	57.9	65	59.6	*0.785*
	Right	54	48.6	46	35.1		56	42.1	44	40.4	
AJCC Stage	I	6	5.4	11	8.4	*0.309*	7	5.3	10	9.2	*0.127*
	II	36	32.4	31	23.7		35	26.3	32	29.4	
	III	29	26.1	44	33.6		36	27.1	37	33.9	
	IV	40	36.0	45	34.4		55	41.4	30	27.5	
Pathology	Adenocarcinoma	102	91.9	129	98.5	*0.097*	124	93.2	107	98.2	*0.298*
	Carcinoma	1	.9	0	.0		1	.8	0	.0	
	Mucinous adenocarcinoma	7	6.3	2	1.5		7	5.3	2	1.8	
	Signet ring cell carcinoma	1	.9	0	.0		1	.8	0	.0	
T4	Not	60	65.9	73	67.0	*0.877*	65	60.7	68	73.1	*0.064*
	Yes	31	34.1	36	33.0		42	39.3	25	26.9	
Grade	Low (G1, G2)	95	88.8	120	93.0	*0.255*	112	87.5	103	95.4	*0.034*
	High (G3, G4)	12	11.2	9	7.0		16	12.5	5	4.6	
Lymphovascular emboli	No	80	76.9	94	75.2	*0.761*	93	75.0	81	77.1	*0.705*
	Yes	24	23.1	31	24.8		31	25.0	24	22.9	
SHP-1	Weak	111	100.0	0	.0	-	69	51.9	42	38.5	*0.038*
	Strong	0	.0	131	100.0		64	48.1	67	61.5	
E-cadherin	Weak	69	62.2	64	48.9	*0.038*	133	100.0	0	.0	-
	Strong	42	37.8	67	51.1		0	.0	109	100.0	

### Loss of p-STAT3^Tyr705^ expression is associated with regorafenib-suppressed mesenchymal phenotype

**Figure 2:**
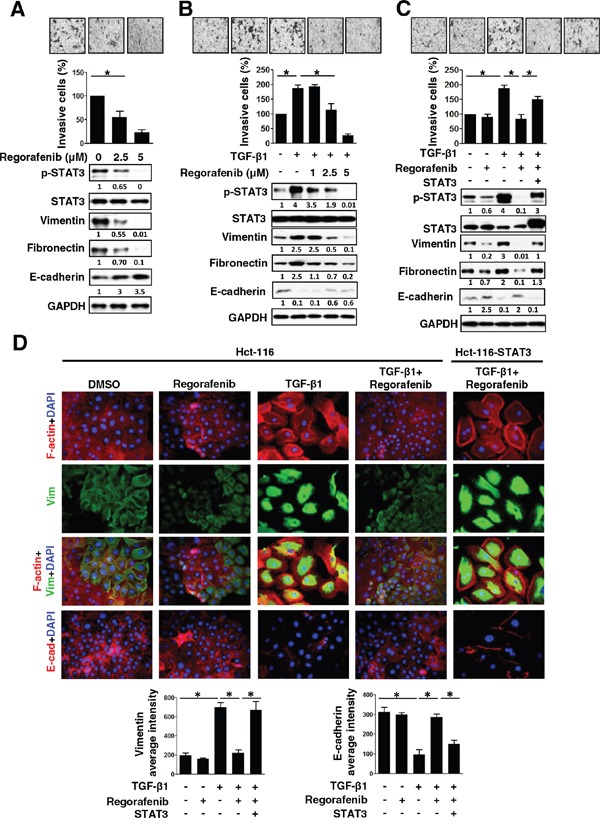
The inhibition of mesenchymal characteristics by regorafenib is dependent on p-STAT3Tyr705 down-regulation **A.** Western blotting of p-STAT3^Tyr705^, STAT3, mesenchymal (vimentin and fibronectin) and epithelial (E-cadherin) markers in SW480 cells 24 h after treatment with dose-escalation of regorafenib. GAPDH was used as a loading control. The invasive capability of cells was measured. The results are shown as mean ± SD of three independent experiments (*, *P* < 0.05). Data are presented as a percentage relative to control SW480 cells. **B.** Western blotting was used to detect the expression of p-STAT3^Tyr705^, STAT3, vimentin, fibronectin and E-cadherin in Hct-116 cells 24 h after either co-treatment with TGF-β1 (10 ng/ml) and/or the indicated dose of regorafenib (1, 2.5, 5 μM). GAPDH was used as a loading control. Invasion assay was performed in cells. The results are shown as mean ± SD of three independent experiments (*, *P* < 0.05). Data are presented as the percentage relative to control Hct-116 cells. **C.** Western blotting of p-STAT3^Tyr705^, STAT3, vimentin, fibronectin and E-cadherin in STAT3-overexpressing Hct-116 cells 24 h after either co-treatment with TGF-β1 (10 ng/ml) and/or regorafenib (2.5 μM). GAPDH was used as a loading control. Invasion assay was performed in these cells. The results are shown as mean ± SD of three independent experiments (*, *P* < 0.05). Data are presented as the percentage relative to control Hct-116 cells. **D.** Immunofluorescence microscopy analysis of rhodamine phalloidin-stained F-actin, DAPI-stained nuclei, vimentin and E-cadherin in the cells shown in (C). Image analysis of vimentin and E-cadherin was quantified by two independent experiments. (*, *P* < 0.05)

Regorafenib is a multitargeted tyrosine kinase (TKI) and the first approved treatment for metastatic CRC [2, 3]. Recently, we reported that sorafenib can significantly inhibit TGF-β1-induced EMT and invasion by SHP-1-dependent STAT3 inhibition [14]. Next, we examined whether regorafenib exerts anti-EMT effects by the loss of p-STAT3^Tyr705^ expression. As shown in Figure 2A, dose-escalation of regorafenib-treated SW480 cells with mesenchymal characteristics showed a gradual decrease in invasive capability along with the decreased expression of p-STAT3^Tyr705^ and mesenchymal markers (vimentin and fibronectin) but had an increased expression of E-cadherin, an epithelial marker. Moreover, when dose-escalation of regorafenib was used to treat epithelial-like cells Hct-116, it resulted in an increase of E-cadherin expression inhibited by TGF-β1. However, it caused significant abolishment of invasive capability as well as decreasing the levels of p-STAT3^Tyr705^ and mesenchymal markers (vimentin and fibronectin) that were increased by TGF-β1 (Figure 2B). The morphological characteristics of TGF-β1-treated Hct-116 cells showed the obvious lost cell-cell contacts and displayed dramatic extensively flattened and elongated leading-trailing mesenchymal morphology along with the increased expression of vimentin and F-actin but concomitant inhibition of E-cadherin expression (Figure 2D). Most notably, these effects were further antagonized by regorafenib and resulted in the acquisition of epithelial-like features, including F-actin-stained cell-cell contacts and increased E-cadherin expression, but reduced the level of vimentin (Figure 2D). These results therefore suggest that the pharmacological mechanism by which regorafenib abolishes the mesenchymal characteristics of CRC cells is likely through the loss of p-STAT3^Tyr705^. We therefore investigated whether STAT3 overexpression rescues the epithelial phenotype in Hct-116 cells co-treated with TGF-β1 and regorafenib and finally enables the occurrence of EMT. Importantly, overexpression of STAT3 in TGF-β1 and regorafenib co-treated Hct-116 cells not only enhanced the expression of p-STAT3^Tyr705^ and mesenchymal markers (vimentin and fibronectin) but also promoted the invasion and exhibited the mesenchymal morphologic characteristics including higher vimentin but lower E-cadherin expression, compared with Hct-116 cells co-treated with TGF-β1 and regorafenib (Figure 2C and 2D). Our data, therefore, provide evidence to identify that regorafenib serves as an anti-EMT therapeutic drug in CRC by antagonizing p-STAT3^Tyr705^ signaling.

### Regorafenib-enhanced SHP-1 tyrosine phosphatase activity is required for the blockage of EMT through p-STAT3^Tyr705^ downregulation

**Figure 3:**
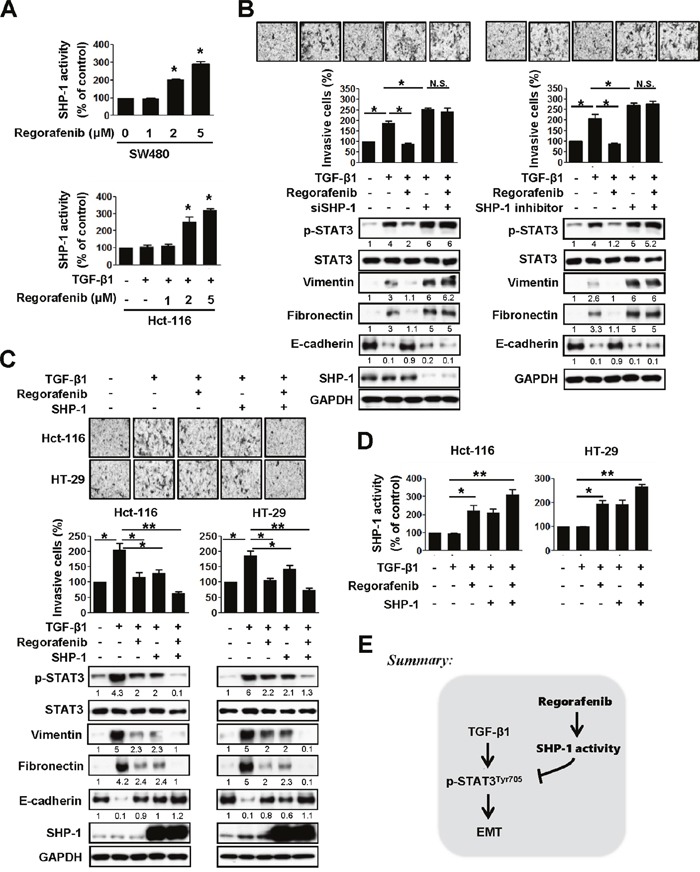
Abolishment of the effects of TGF-β1 on p-STAT3Tyr705 and EMT by regorafenib is through the enhancement of SHP-1 activity, resulting in down-regulation of p-STAT3Tyr705 **A.** *Upper panels*, SHP-1 activity was measured in SW480 cells treated with dose-escalation of regorafenib for 24 h. *Lower panels*, SHP-1 activity was measured in the Hct-116 cells either co-treated with TGF-β1 (10 ng/ml) and/or the indicated dose of regorafenib for 24 h. The results are shown as mean ± SD of three independent experiments (*, *P* < 0.05). Data are presented as the percentage relative to control SW480 or Hct-116 cells. **B.** *Left panels*, western blotting of p-STAT3^Tyr705^, STAT3, vimentin, fibronectin and E-cadherin in TGF-β1-treated Hct-116 cells 24 h after either co-treatment with or without SHP-1 siRNA (25 nM) and/or regorafenib (2.5 μM). *Right panels*, western blotting of p-STAT3^Tyr705^, STAT3, vimentin, fibronectin and E-cadherin in TGF-β1-treated Hct-116 cells 24 h after either co-treatment with or without SHP-1 inhibitor (20 nM) and/or regorafenib (2.5 μM). GAPDH was used as a loading control. Invasion assay was performed in these cells shown in *left* and *right panels*. The results are shown as mean ± SD of three independent experiments (*, *P* < 0.05; N.S., non-significant). Data are presented as the percentage relative to control Hct-116 cells. **C.** Western blotting of p-STAT3^Tyr705^, STAT3, vimentin, fibronectin and E-cadherin in SHP-1-overexpressed Hct-116 and HT-29 cells 24 h after either co-treatment with TGF-β1 (10 ng/ml) and/or regorafenib (2.5 μM). GAPDH was used as a loading control. The invasive capability was measured in these cells. The results are shown as mean ± SD of three independent experiments (*, *P* < 0.05, **, *P* < 0.01). Data are presented as a percentage relative to the control Hct-116 or HT-29 cells. **D.** SHP-1 activity was performed in cells shown in (C). The results are shown as mean ± SD of three independent experiments (*, *P* < 0.05, **, *P* < 0.01). Data are presented as a percentage relative to control Hct-116 or HT-29 cells. **E.** A mechanistic representation showing that regorafenib prevents TGF-β1-induced EMT mediated through regorafneib-enhanced SHP-1 activity that effectively downregulates p-STAT3^Tyr705^ expression.

Because SHP-1 tyrosine phosphatase activity could negatively regulate HCC EMT by mediating the inactivation of STAT3 [14], we then evaluated whether SHP-1 tyrosine phosphatase activity is involved in the effects of regorafenib on reducing TGF-β1-induced p-STAT3^Tyr705^, which is required for EMT. We found that regorafenib significantly increased SHP-1 activity in SW480 cells in a dose-dependent manner (Figure 3A, *upper panels*). Regorafenib-enhanced SHP-1 activity was also seen in Hct-116 cells co-treated with TGF-β1 and dose-escalation of regorafenib, compared with TGF-β1-treated cells (Figure 3A, *lower panels*). Next, in order to know whether regorafenib reduced TGF-β1-induced p-STAT3^Tyr705^ expression is SHP-1-dependent, we employed siRNA to deplete SHP-1 and found that knockdown of SHP-1 in TGF-β1-treated Hct-116 cells significantly increased both the levels of p-STAT3^Tyr705^ and mesenchymal markers as well promoting invasive capability, compared with TGF-β1-treated cancer cells (Figure 3B, *left panels*). However, their expression levels were not further altered in SHP-1-depleted cancer cells co-treated with TGF-β1 and regorafenib (Figure 3B, *left panels*). These observations were further confirmed by using SHP-1 phosphatase-specific inhibitor (PTPIII) to inhibit SHP-1 activity as shown in Figure 3B, *right panels*. These findings indicate that SHP-1 plays a key role in mediating the effect of regorafenib on reducing TGF-β1-induced p-STAT3^Tyr705^ activation. In contrast, overexpression of SHP-1 significantly reduced the effect of TGF-β1 on the levels of p-STAT3^Tyr705^ and mesenchymal markers in Hct-116 and HT-29 cells and the treatment of regorafenib further enhanced these effects by SHP-1 overexpression (Figure 3C). Most importantly, regorafenib upregulated the activity of SHP-1 significantly in SHP-1-overexpressed Hct-116 and HT-29 cells treated with TGF-β1 (Figure 3D). Taken together, these results clearly demonstrate that regorafenib abolished the effects of TGF-β1 on p-STAT3^Tyr705^ and EMT was through the enhancement of the SHP-1 activity, resulting in down-regulation of p-STAT3^Tyr705^ (Figure 3E).

### Regorafenib directly activates SHP-1 by potently relieving the autoinhibited N-SH2 domain of SHP-1 to inhibit p-STAT3^Tyr705^ level and EMT

**Figure 4:**
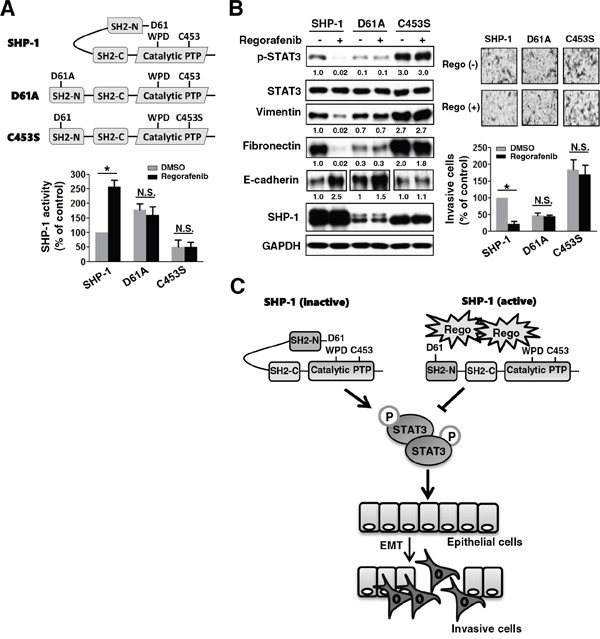
Regorafenib enhances SHP-1 activity through potently relieving the autoinhibition of SHP-1, which reinforces the suppressive effect on p-STAT3Tyr705 and mesenchymal characteristics **A.** *Upper panels*, a schematic representation of wild-type and mutant-type SHP-1 including D61A and C453S carrying constitutive and dead activity of SHP-1, respectively. *Lower panels*, SHP-1 activity was assessed in the wild-type and mutant-type (D61A and C453S) SHP-1-transfected SW480 cells 2 days after transfection with these plasmids. The results are shown as mean ± SD of three independent experiments made in triplicate. (*, *P* < 0.05; N.S., non-significant) **B.** *Left panels*, western blotting of SHP-1, p-STAT3^Tyr705^, STAT3, and EMT markers in the wild-type and mutant-type (D61A and C453S) SHP-1-transfected SW480 cells 24 h after treatment with or without regorafenib at 2.5 μM. *Right panels*, the invasive capability was measured in the cells shown in the *left panels* of (B). The results are shown as mean ± SD of three independent experiments, made in triplicate. (*, *P* < 0.05; N.S., non-significant) **C.** Regorafenib suppressed TGF-β1-induced EMT and invasion in CRC which was mediated through regorafenib-enhanced SHP-1 activity. Regorafenib had the potential to dock to the inhibitory N-SH2 domain and the catalytic PTP domain of SHP-1, resulting in the direct relief of autoinhibition of SHP-1. The activity of SHP-1 tyrosine phosphatase specifically increased the susceptibility to p-STAT3^Tyr705^, which antagonized the EMT pathway.

To further investigate the druggable mechanism through which regorafenib negatively regulates EMT by increasing SHP-1 tyrosine phosphatase activity, we transfected mesenchymal cells SW480 with wild-type or mutant SHP-1 (D61A and C453S). In SHP-1, intramolecular inhibition occurs through association of the N-SH2 domain with the PTP catalytic domain which is stabilized by a salt bridge between Asp61 (D61) and Lys362 (Figure 4A, *upper panels*). The D61A point mutant mimics the open conformation of SHP-1 and serves as a constitutive activator. C453S is the catalytic-dead mutant of SHP-1 (Figure 4A, *upper panels*). The D61A mutant SHP-1 showed an increase in SHP-1 tyrosine phosphatase activity (Figure 4A, *lower panels*) but displayed a marked decrease in p-STAT3^Tyr705^ and mesenchymal markers vimentin and fibronectin, compared with wild-type and C453S mutant SHP-1 (Figure 4B, *left panels*). Importantly, regorafenib significantly increased SHP-1 tyrosine phosphatase activity in wild-type SHP-1-transfected but not in D61A or C453S mutant SHP-1-transfected SW480 cells (Figure 4A, *lower panels*), suggesting that regorafenib increases SHP-1 tyrosine phosphatase activity through the direct disruption of the interaction between the N-SH2 domain and the PTP catalytic domain of SHP-1 that further relieves the autoinhibition of SHP-1. In addition, wild-type SHP-1-transfected SW480 cells showed a marked decrease in expression of p-STAT3^Tyr705^ and mesenchymal markers (vimentin and fibronectin) but had a significant increase expression of E-cadherin after regorafenib treatment (Figure 4B, *left panels*). These cells displayed a significant reduction in invasive capability (Figure 4B, *right panels*). However, this observation was not seen in D61A or C453S mutant SHP-1-transfected cells following treatment with regorafenib (Figure 4B). Our data therefore identifies SHP-1 as a druggable target of regorafenib verified by the evidence that regorafenib pharmacologically increases SHP-1 activity through relieving the inhibitory N-SH2 domain of SHP-1, leading to reduced p-STAT3^Tyr705^ level and eventual suppression of EMT in CRC cells (Figure 4C).

### Regorafenib exerts significant anti-metastatic growth of CRC *in vivo*

**Figure 5:**
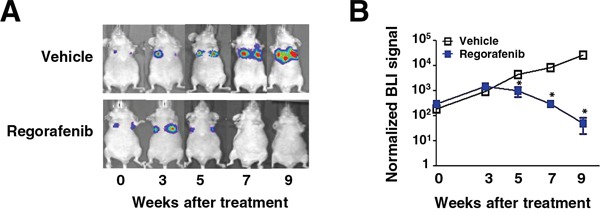
Regorafenib suppresses lung metastatic outgrowth of CRC cells *in vivo* **A.** Representative BLI images of animals in each experimental group at the indicated time points after receiving the indicated drug treatment. **B.** Normalized BLI signals of lung metastatic outgrowth in mice (n = 5) receiving regorafenib (10 mg/kg/day) or vehicle, daily, when metastatic growth was initially seen in the lung after tail vein injection with *Luc2*-expressing SW480 cells (2 × 10^6^). *Points*, mean (n = 5); *bars*, SEM. *, *P* < 0.05.

Next, in order to evaluate the therapeutic efficacy of regorafenib on metastasis *in vivo*, highly metastatic luciferase (Luc) 2-expressing SW480 cells (2 × 10^6^) with mesenchymal characteristics were injected into the tail vein of nude mice and then treated with regorafenib (p.o.,10 mg/kg/day) or vehicle when cells initially showed metastatic growth in the lung monitored by bioluminescent imaging. Importantly, mice receiving regorafenib treatment exhibited a significant reduction in the lung metastatic outgrowth of SW480 cells in comparison with vehicle treatment (Figure 5A and 5B). The data above not only demonstrate that regorafenib is efficacious at curbing metastatic growth of mesenchymal CRC cells *in vivo* but also further support our *in vitro* data in suggesting that regorafenib targets SHP-1-dependent STAT3 inactivation during CRC EMT and metastasis.

## DISCUSSION

The cause of the mCRC emergence is highly complex and heterogeneous, and usually results in significant mortality in patients when resistance occurs from the currently available multiple lines of targeted therapies, such as anti-EGFR antibodies cetuximab and panitumumab, and VEGF inhibitors bevacizumab and aflibercept. Recently, regorafenib became the first approved pharmacotherapy for mCRC, serving as a TKI that blocks key molecular markers involved in oncogenesis, angiogenesis and metastasis. Here, we provide further insight into the relationship between the PTPase SHP-1-targeted therapy by regorafenib and CRC EMT reported to be involved in cancer invasion/metastasis. We provide evidence that regorafenib curbs TGF-β1-induced EMT/invasion *in vitro* by activating PTPase SHP-1-dependent p-STAT3^Tyr705^ suppression. Increasing PTPase SHP-1 activity by gain-of-function overexpression of wild-type SHP-1 further re-enforces the negative effects of regorafenib on TGF-β1-induced p-STAT3^Tyr705^ and EMT/invasion. Conversely, decreasing PTPase SHP-1 activity significantly restores the negative effects of regorafenib on p-STAT3^Tyr705^ and EMT/invasion as proved by SHP-1 inhibitor or siRNA-mediated SHP-1 depletion. Together, we have a novel discovery that regorafenib exerts anti-EMT potency by serving a SHP-1 agonist to activate the activity of SHP-1, as an EMT-associated suppressor. Our *in vivo* data also indicate that regorafenib suppresses metastatic outgrowth of SW480 mesenchymal cells by tail-vein injection metastatic model. However, the sufficient data from *in vivo* animal study is still lack. Therefore, much more attention should be paid when generating the orthotopic metastatic model that implantation of cells into the spleen and then to see the liver metastasis. Using this methodology in place of examining the lung metastasis by tail-vein injection metastatic model will be a reliable approach to further support our *in vitro* findings.

Mechanistically, regorafenib effectively increases SHP-1 activity because of the docking potential of regorafenib into the N-SH2 and catalytic PTP domain of SHP-1 that relieves the autoinhibiton of SHP-1, resulting in the reduction of both p-STAT3^Tyr705^ and EMT/invasion induced by TGF-β1. Interestingly, our previous data indicated that sorafenib, an analog of regorafenib, shows significant anti-EMT activity *in vitro* and *in vivo* in a HCC preclinical model *via* directly activating PTPase SHP-1-dependent p-STAT3^Tyr705^ suppression [14]. Together, these findings firmly confirm that the negative regulatory PTPase SHP-1 is an EMT/metastasis-suppressing gene, whose activity can be pharmacologically activated by TKIs that act not only as STAT3 inhibitors, but also as SHP-1 agonists, such as regorafenib and sorafenib.

Ample evidence suggests that the loss of E-cadherin is necessary to induce EMT/metastasis of cancer cells [14–17]. On the other hand, restoration of E-cadherin expression enhances intercellular adhesion, and suppresses the EMT/metastasis [14, 21, 22]. Here, through assay of 243 CRC patients by a Kaplan-Meier analysis, the median OS was not available in patients with strong expression both in SHP-1 and E-cadherin. In contrast, patients with weak expression of both SHP-1 and E-cadherin had a more significant reduction in median OS than others. These observations imply that patients with highly levels of SHP-1-E-cadherin expression display longer survival. In addition, the clinical data reveal that a positive correlation significantly existed between SHP-1 and E-cadherin expression in CRC tissues. Interestingly, the expression of E-cadherin, but not SHP-1, had an inverse correlation with histological grading. These data suggest that SHP-1 may not be the only upstream positive regulator of E-cadherin. Other upstream regulators may also positively control the expression of E-cadherin to finally establish the adherens junctions. For example, GSK-3β positively regulates E-cadherin by negatively controlling the stability of SNAIL/SLUG, as the repressors of E-cadherin [23]. In addition, MicroRNA-200a has been reported to function as an EMT-associated tumor suppressor to upregulate E-cadherin by downregulating ZEB1/2, as the repressors of E-cadherin [24]. Therefore, E-cadherin, but not SHP-1, negatively correlates with histological grading may due to the reason that other upstream regulators of E-cadherin are essential to induce the expression of E-cadherin, which serves as a direct downstream effector and significantly associates with the low-grading cells with well differentiation. SHP-1 is one of the upstream positive regulators of E-cadherin and not an independent effector to directly associate with histological grading in the present study. Collectively, these clinical data are further confirming the *in vitro* findings that SHP-1 is an EMT-associated suppressor to upregulate E-cadherin by suppressing p-STAT3^Tyr705^.

In summary, this is the first report showing a novel link between regorafenib and CRC EMT. Regorafenib guides anti-EMT/metastasis therapy *in vitro* and *in vivo*, through the activation of PTPase SHP-1-dependent p-STAT3^Tyr705^ suppression. Regorafenib with potent anti-EMT activity serves as a SHP-1 agonist by the relief of autoinhibition in SHP-1. Of note, SHP-1 is identified as not only as a suppressor of EMT/metastasis but also as a pharmacologic target of regorafenib. Further, we establish a novel link between the SHP-1-E-cadherin axis and overall survival of CRC pateints. The findings of this study may guide the development of alternative PTPase-targeted therapy for mCRC patients who have failed multiple lines of therapy, and shows that anti-EMT potency in regorafenib significantly activates PTPase SHP-1-dependent STAT3 inactivation, and might provide a straightforward pharmacotherapy for mCRC.

## MATERIALS AND METHODS

### Cell culture

CRC cell lines, including Hct-116, HT-29 and SW480 cell lines were maintained in RPMI1640 medium supplemented with 10% FBS, 100 units/ml of penicillin and streptomycin (Invitrogen, Carlsbad, CA, USA), and then incubated at 37°C in a humidified 5% CO_2_ atmosphere.

### Reagents and plasmids

Recombinant human TGF-β1 was purchased from R&D Systems (Minneapolis, MN). Regorafenib was kindly provided by Bayer HealthCare Pharmaceuticals. For *in vitro* studies, regorafenib was dissolved in dimethyl sulfoxide and then added to the cells maintained in RPMI 1640 medium without FBS. For *in vivo* animal study, regorafenib was suspended in Kolliphor (JT Baker). SHP-1 inhibitor (PTP III) was purchased from Calbiochem. Smart-pool siRNA, including control (D-001810-10), SHP-1 (PTPN6, L-009778-00-0005) were all purchased from Dharmacon (Chicago, IL). Plasmids of human wild-type STAT3 and SHP-1 (PTPN6) were encoded by pCMV6 vector with myc-tag. For mutant-type SHP-1 expression, we generated a D61A plasmid that changed aspartic acid at 61 to an alanine residue, which was cloned into pCMV6-Entry vector. Mutant-type SHP-1 C453S was encoded by pJ3-SHP-1 plasmid purchased from Addgene plasmid repository (http://www.addgene.org/). Smart-pool siRNA, including control (D-001810-10), SHP-1 (PTPN6, L-009778-00-0005) were purchased from Dharmacon (Chicago, IL). These plasmids or siRNA were subsequently transfected into cells by using Lipofectamine 2000 reagent (Invitrogene, CA).

### SHP-1 phosphatase activity

The cellular protein extracts were incubated with anti-SHP-1 antibody in immunoprecipitation buffer overnight. Protein G-Sepharose 4 Fast flow (GE Healthcare Bio-Science, NJ) was added to each sample, followed by incubation for 3 h at 4°C with rotation. A RediPlate 96 EnzChekR Tyrosine Phosphatase Assay Kit (R-22067) was used for SHP-1 activity assay (Molecular Probes, Invitrogen, CA).

### *In vitro* invasion assay

The invasive capability of cells was examined using polycarbonate transwell filters containing 8-μm pores (Corning Coster, Cambridge, MA). Cells (3 × 10^5^) seeded in serum-free medium on the upper side of the chamber coated with Matrigel (BD Biosciences, Bedford, MA, USA). The cells were allowed to migrate toward the lower chamber containing media supplemented with 10% FBS. After 24-hours, cells on the lower side of the membrane were fixed, stained with crystal violent and then counted. The average number of the crystal violent stained-cells was calculated by three independent experiments. The data of invasive cells is presented as a percentage normalized to the control according to each experiment.

### Immunofluorescent staining

Cells seeded on a coverslip were fixed in 4% paraformaldehyde, permeabilized with 0.25% Triton X-100, and then blocked with 5% bovine serum albumin (BSA). After that, primary antibodies were incubated against vimentin (Abcam), E-cadherin (Abcam). Rhodamine phalloidin (Invitrogen) and DAPI were used for F-actin and nuclear staining, respectively. The average intensity of vimentin and E-cadherin was determined by densitometry Image J software. Statistical analyses were performed using SPSS 13.0 for Windows (SPSS Inc., Chicago, IL, USA).

### Western blotting

Whole-cell lysates were made in RIPA buffer and subjected to SDS-PAGE, transferred onto a polyvinylidene difluoride membrane (Millipore, Billerica, MA, USA) and incubated with primary antibody, and then incubated with horseradish peroxidase-conjugated secondary antibodies. Specific proteins were detected using enhanced chemiluminescence reagent. The primary antibodies including p-STAT3^Tyr705^, STAT3, SHP-1, Vimentin, Fibronectin, E-cadherin and GAPDH were purchased from Abcam (Cambridge, MA). For quantification of protein levels on chemiluminescent western blots, densitometry Image J software was employed. Statistical analyses were performed using SPSS 13.0 for Windows (SPSS Inc., Chicago, IL, USA).

### *In vivo* animal study

Luciferase (*Luc*) 2-expressing SW480 cells (2 × 10^6^) suspended in 100 μL PBS were injected into the tail vein of 6-week-old nude mice. Mice were randomly grouped into regorafenib- (p.o., 10 mg/kg/day) or a vehicle-treatment group when cells were initially observed to metastasize to the lung and monitored by bioluminescent imaging (BLI). To evaluate the therapeutic efficacy of regorafenib on the metastatic outgrowth of SW480 cells in lungs, BLI was used for regorafenib- or vehicle-treated mice on weeks 0, 3, 5, 7, and 9.

### Patient specimens

Patients with colorectal cancer, determined according to the World Health Organization criteria, were enrolled from January 2002 through January 2014 and classified according to the American Joint Committee on Cancer (AJCC) staging system (Version 6). Clinical data were obtained from the cancer registry. Overall survival (OS) was defined as the time from primary resection to death from cancer. The follow-up period ended in January 2014, or at the time of death of the patient. Left colon cancer was defined as a malignancy in the splenic flexure, descending colon, sigmoid colon, rectosigmoid colon and rectum.

### Immunohistochemistry

For tissue microarray (TMA) and immunohistochemistry (IHC), the procedures followed our previous methods and the manufacturer's instructions [20]. SHP-1 and E-cadherin antibodies were purchased from Abcam (Cambridge, MA). Omission of the primary antibody served as a negative control. Immunopositive results were evaluated by 2 pathologists. The intensity of stained cells was scored as 0, 1, 2, or 3. Percentages of stained cells were counted. A final immunohistochemical score (H-score) was calculated by summing the products of the staining intensities (0–3) and distributions (0%–100%). H-scores ranged from 0 to 300. An H-score equal to or greater than 150 was defined as strongly positive for SHP-1 staining; all others were scored as weakly positive. An H-score greater than 150 was defined as strongly positive for E-cadherin staining; all others were scored as weakly positive.

### Statistical and survival analysis

Quantitative data are presented as the mean ± standard deviation (SD) from three independent experiments. The *t* test was used to compare age distribution. The correlations between clinicopathological variables and immunopositivity were analyzed using the χ^2^ test or Fisher's exact test. Survival was estimated using the Kaplan-Meier method. A 2-sided P value of less than 0.05 was regarded as statistically significant. SPSS software (version 16.00, SPSS, Chicago, IL) was used for all the statistical analyses.

## SUPPLEMENTARY MATERIALS FIGURE


