# Supplementary material for: Regorafenib (Stivarga) pharmacologically targets epithelial-mesenchymal transition in colorectal cancer
Source: Oncotarget. 2016 Aug 26;7(39):64136–47. doi: 10.18632/oncotarget.11636 (PMC5325431; doi:10.18632/oncotarget.11636)
Supplement: Supplementary file 1 [file oncotarget-07-64136-s001.pdf]

Regorafenib (Stivarga) pharmacologically targets epithelial-mesenchymal transition in colorectal cancer

SUPPLEMENTARY FIGURE

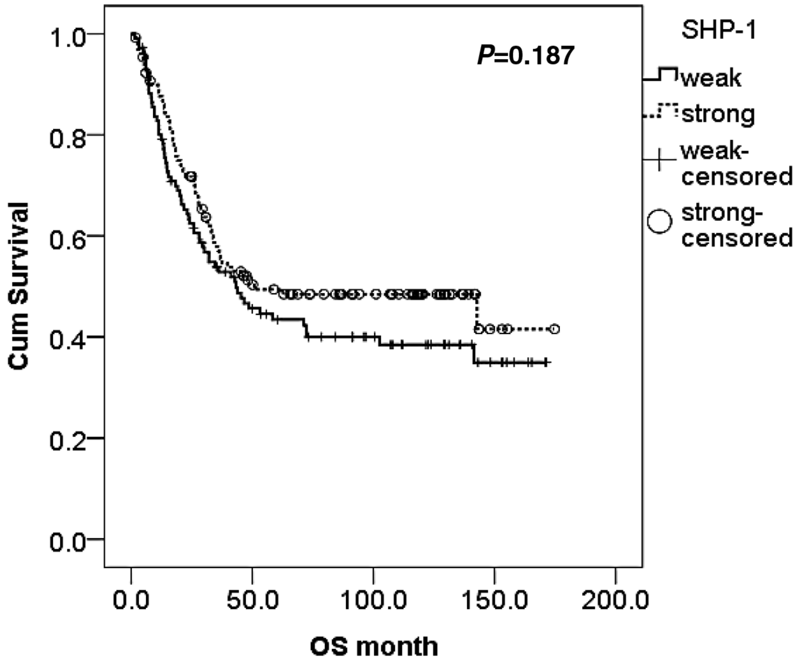

Supplementary Figure S1: The survival curves of CRC patients with and without upregulated SHP-1 (calculated using the Kaplan–Meier method). No significant correlation between median overall survival (OS) and SHP-1 expression (*P*=0.187).
